# A Grade 1, Metastatic, Well-Differentiated Neuroendocrine Tumor Presenting as Closed-Loop Small Bowel Obstruction With Ischemic Bowel

**DOI:** 10.7759/cureus.107524

**Published:** 2026-04-22

**Authors:** Rodrigo Furlan Silva Fabri, Rodolfo Myronn De Melo Rodrigues, Nawar Hakim, Lela Ruck

**Affiliations:** 1 Internal Medicine, Paul L. Foster School of Medicine, Texas Tech University Health Sciences Center El Paso, El Paso, USA; 2 Pathology, Paul L. Foster School of Medicine, Texas Tech University Health Sciences Center El Paso, El Paso, USA

**Keywords:** appendectomy, bowel wall thickening, emergency exploratory laparotomy, ischemia, luminal narrowing, mesenteric lymph nodes, mesenteric vascular compromise, metastatic well-differentiated neuroendocrine tumor, primary anastomosis, stellate mesenteric mass

## Abstract

Small bowel neuroendocrine tumors (NETs) are typically indolent neoplasms that often present with vague abdominal symptoms and are frequently diagnosed only after regional or metastatic spread has occurred. Mesenteric nodal metastases may provoke a desmoplastic reaction resulting in bowel tethering, luminal narrowing, mesenteric vascular compromise, obstruction, and intestinal ischemia. We report the case of a 49-year-old man with no prior abdominal surgery who presented with three months of intermittent abdominal pain followed by abrupt worsening to diffuse severe abdominal pain and emesis. Computed tomography demonstrated small bowel obstruction with concern for a closed-loop obstruction at the level of a 2.5 × 1.6 cm mesenteric mass, with associated bowel wall thickening concerning for ischemia. The patient underwent emergency exploratory laparotomy with resection of strangulated small bowel, en bloc resection of a stellate mesenteric mass, primary anastomosis, and appendectomy. Pathology demonstrated mesenteric lymph nodes positive for metastatic well-differentiated NET, grade 1, with extranodal extension and lymphatic space invasion. Tumor cells were positive for synaptophysin, chromogranin, CD56, and CK AE1/AE3, with a Ki-67 index of less than 1%. No definite primary lesion was identified in the resected bowel specimen, but the overall clinicopathologic findings strongly suggested an occult small bowel primary. The postoperative course was uncomplicated, and the patient improved clinically after surgery. This case highlights that even low-grade NETs may first present as a surgical emergency due to mesenteric metastatic disease and fibrosis rather than carcinoid syndrome. In patients with a virgin abdomen, chronic intermittent abdominal pain, weight loss, and an obstructing mesenteric mass, small bowel NET should remain in the differential diagnosis.

## Introduction

Neuroendocrine tumors (NETs) of the small intestine are the most common malignant neoplasms of the small bowel, and their incidence and prevalence have increased over recent decades [1-3]. Although these tumors are often biologically indolent, they frequently present late, often after regional nodal or distant metastatic spread has already occurred [1,2]. 

A distinctive feature of small bowel NETs is the tendency of mesenteric nodal metastases to provoke a desmoplastic fibrotic reaction. This process may result in bowel tethering, luminal narrowing, chronic abdominal pain, obstruction, and mesenteric vascular compromise, including ischemia [4-10].

Many patients initially present with vague and nonspecific abdominal symptoms, which may delay diagnosis. In a subset of cases, however, the first manifestation is an acute surgical complication such as bowel obstruction, perforation, bleeding, or ischemia [4,11,12]. This pattern is clinically important because emergency surgery for small bowel NETs has been associated with less favorable oncologic outcomes compared with elective intervention [11,12].

We present a case of a grade 1 metastatic well-differentiated NET presenting as closed-loop small bowel obstruction with strangulation and ischemic bowel, highlighting the diagnostic challenge posed by months of nonspecific symptoms before abrupt clinical deterioration requiring emergency resection. This case is noteworthy because it illustrates how a biologically low-grade NET may initially present as a surgical emergency caused by mesenteric metastatic fibrosis and bowel ischemia rather than by hormonal symptoms or an elective oncologic workup. 

## Case presentation

A 49-year-old man with no known past medical history, no prior abdominal surgery, and no chronic medication use presented to the emergency department with severe abdominal pain and vomiting. He reported a three-month history of intermittent lower abdominal pain that acutely worsened the night before presentation, becoming constant, diffuse, and severe, rated 10/10. He had one episode of brown emesis. He denied fever, chills, melena, hematochezia, flushing, wheezing, diarrhea, hypotension, or urinary symptoms. He also reported an unintentional weight loss of approximately 20 pounds over the preceding month.

On arrival, he was afebrile and hemodynamically stable. Physical examination showed a soft but minimally distended abdomen with markedly decreased bowel sounds and diffuse lower abdominal tenderness. A firm area was palpated in the upper abdomen just left of the midline. There were no prior surgical scars or hernias. Initial laboratory evaluation was notable for the absence of leukocytosis, electrolyte derangement, or lactic acidosis.

**Figure 1:**
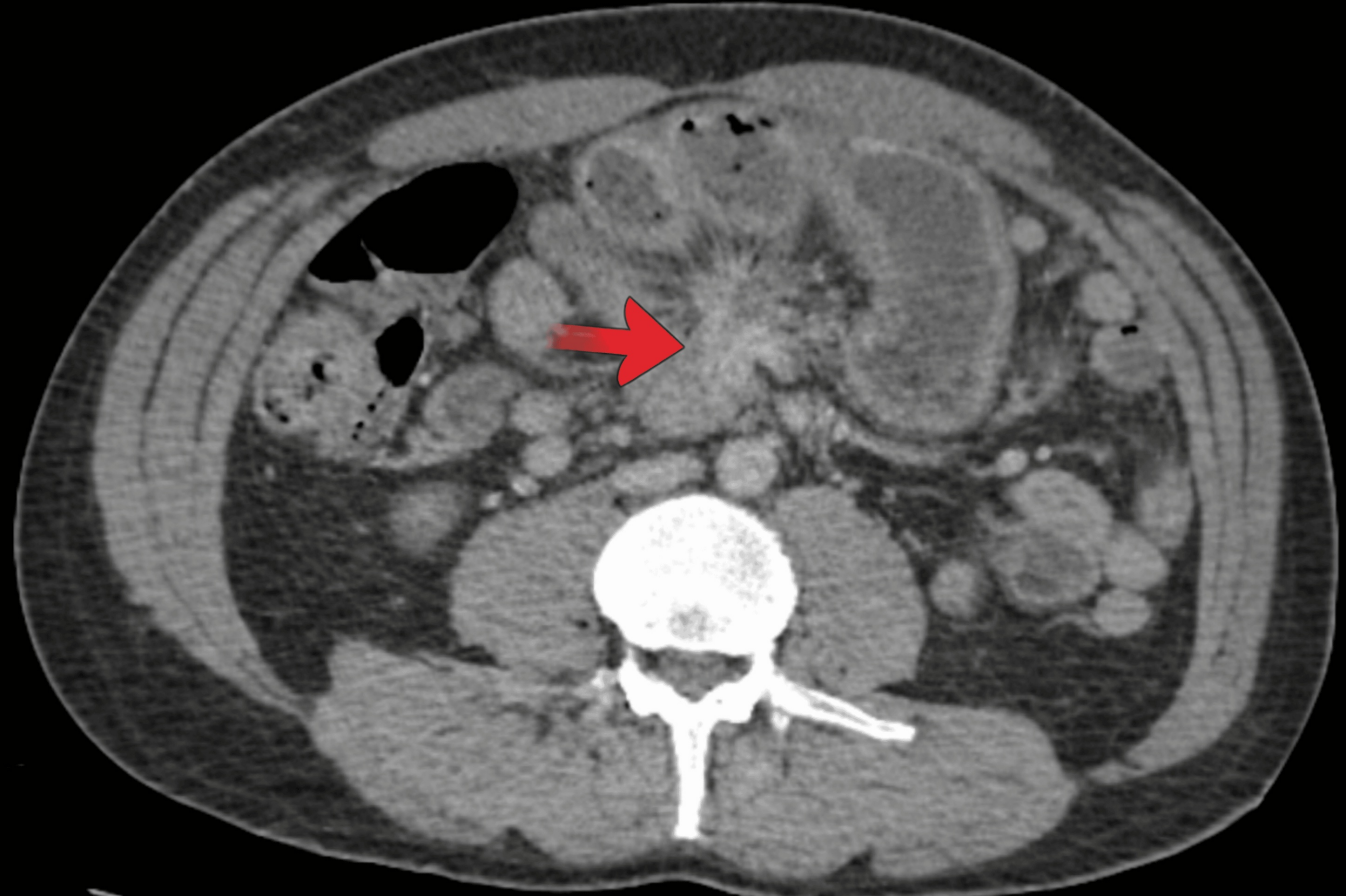
Contrast-enhanced CT of the abdomen and pelvis demonstrating a mesenteric mass (red arrow) at the transition point of small bowel obstruction. Converging mesenteric vessels and surrounding mesenteric stranding are present, findings characteristic of the desmoplastic reaction associated with small bowel neuroendocrine tumors. Adjacent dilated small bowel loops are visible, consistent with a closed-loop obstruction and impending bowel ischemia.

Contrast-enhanced computed tomography (CT) of the abdomen and pelvis demonstrated small bowel obstruction with concern for a closed-loop obstruction at the level of a 2.5 × 1.6 cm mesenteric mass. Mesenteric vessels converged toward the mass, and associated bowel wall thickening and mucosal enhancement raised concern for ischemia. Small-volume pelvic free fluid was present. No liver metastases were identified on this examination (Figure 1). Given the concern for strangulation and evolving bowel compromise, the patient was taken urgently to the operating room.

He underwent exploratory laparotomy with resection of the strangulated small bowel, en bloc resection of a stellate mesenteric mass, primary anastomosis, and appendectomy. Intraoperatively, numerous dusky, thick-walled loops of small bowel were found tightly incarcerated in the midabdomen and tethered by a bleeding stellate mesenteric mass. The liver was palpated, and no gross masses were identified. Approximately 290 cm of proximal viable small bowel was preserved before the diseased segment, while only approximately 15 cm of healthy distal bowel remained proximal to the ileocecal valve at the level of the involved mesentery. The involved segment and mesenteric mass were resected en bloc, followed by primary small bowel anastomosis. Repeat inspection confirmed the viability of the remaining bowel. The appendix was also removed because it appeared dilated and fibrotic.

**Figure 2:**
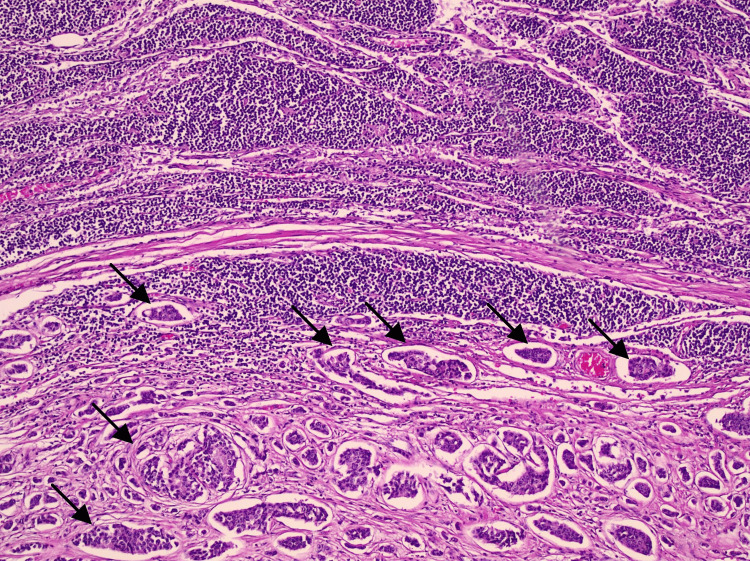
Histopathologic features of metastatic well-differentiated NET in mesenteric lymph node Hematoxylin and eosin (H&E) stain of a mesenteric lymph node demonstrating partial effacement by metastatic well-differentiated NET. The lower half of the image shows replacement of normal nodal architecture by nests and trabeculae of uniform tumor cells with round nuclei and finely stippled (“salt-and-pepper”) chromatin. Arrows highlight areas of tumor infiltration. Original magnification ×100. NET: neuroendocrine tumor

**Figure 3:**
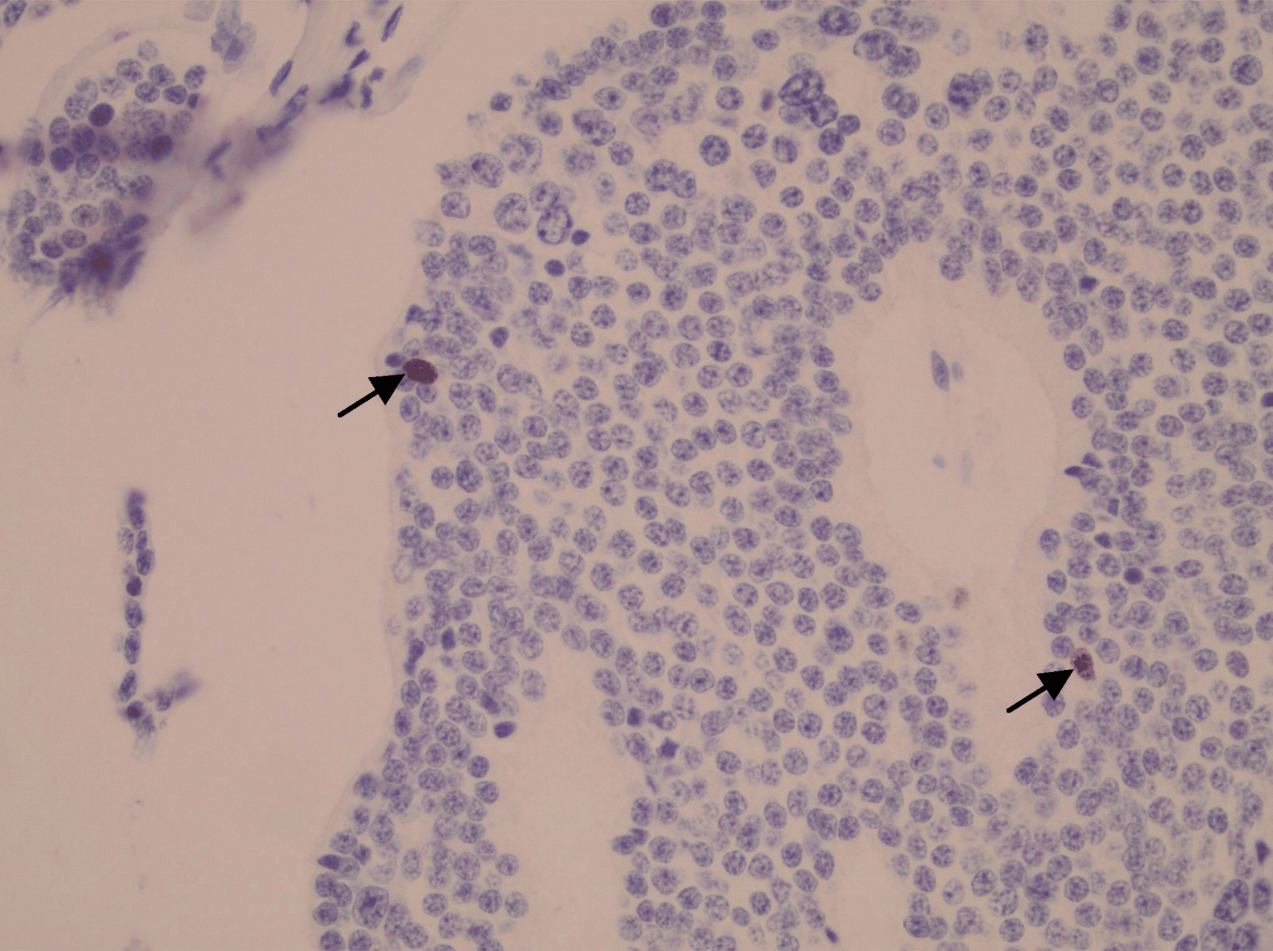
Ki-67 immunohistochemical staining demonstrating low proliferative index in well-differentiated NET Immunohistochemical staining for Ki-67 demonstrating a very low proliferative index, with only rare tumor cell nuclei showing positive staining (arrows). The majority of tumor cells are negative, supporting a low-grade (grade 1) well-differentiated NET. Original magnification ×400. NET: neuroendocrine tumor

**Figure 4:**
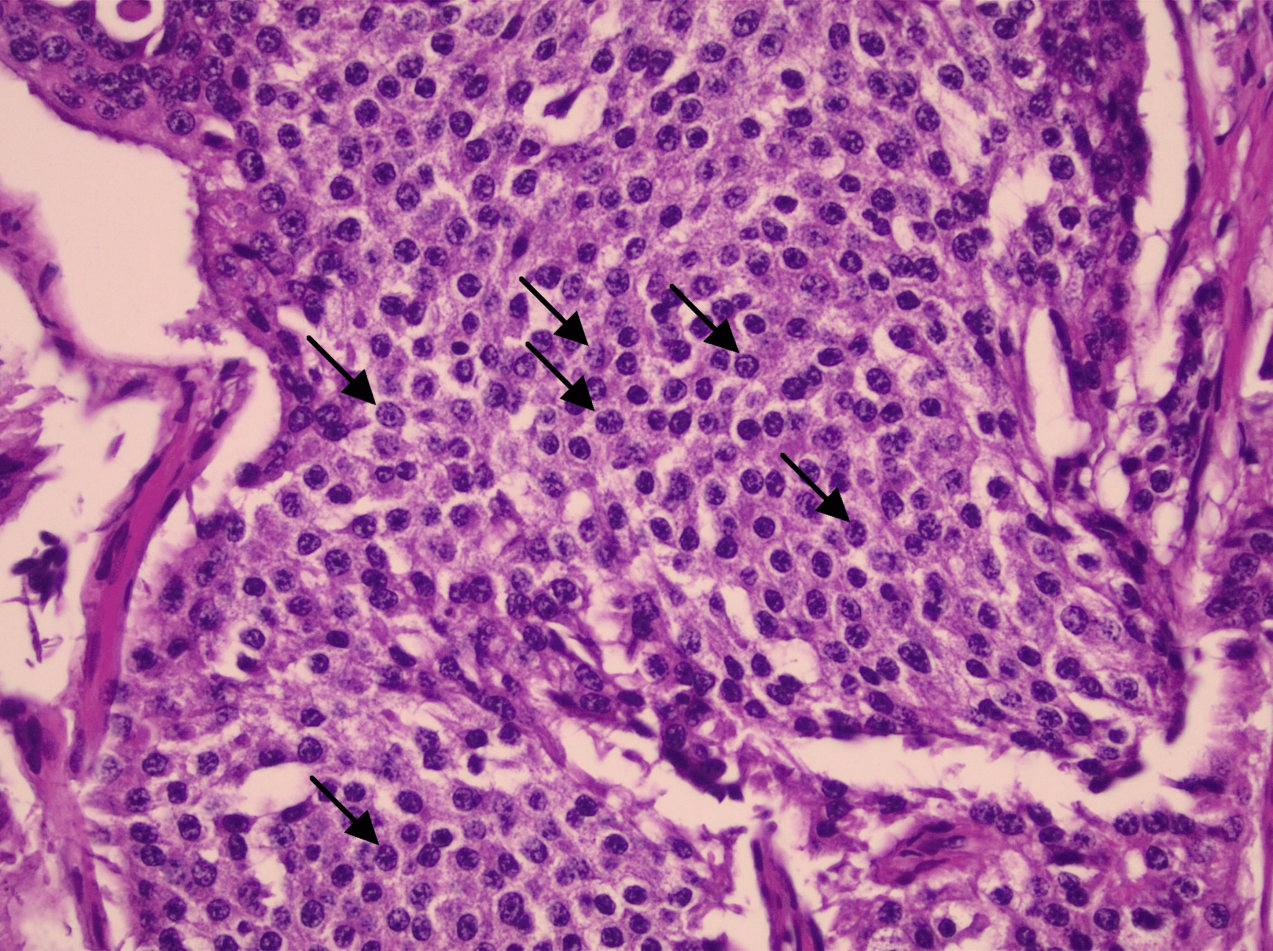
Cytologic features of well-differentiated neuroendocrine tumor with salt-and-pepper chromatin High-power hematoxylin and eosin (H&E) stain demonstrating nests of uniform tumor cells with round to oval nuclei, finely stippled (“salt-and-pepper”) chromatin, and minimal nuclear pleomorphism. Arrows highlight the characteristic chromatin pattern. Original magnification ×400.

**Figure 5:**
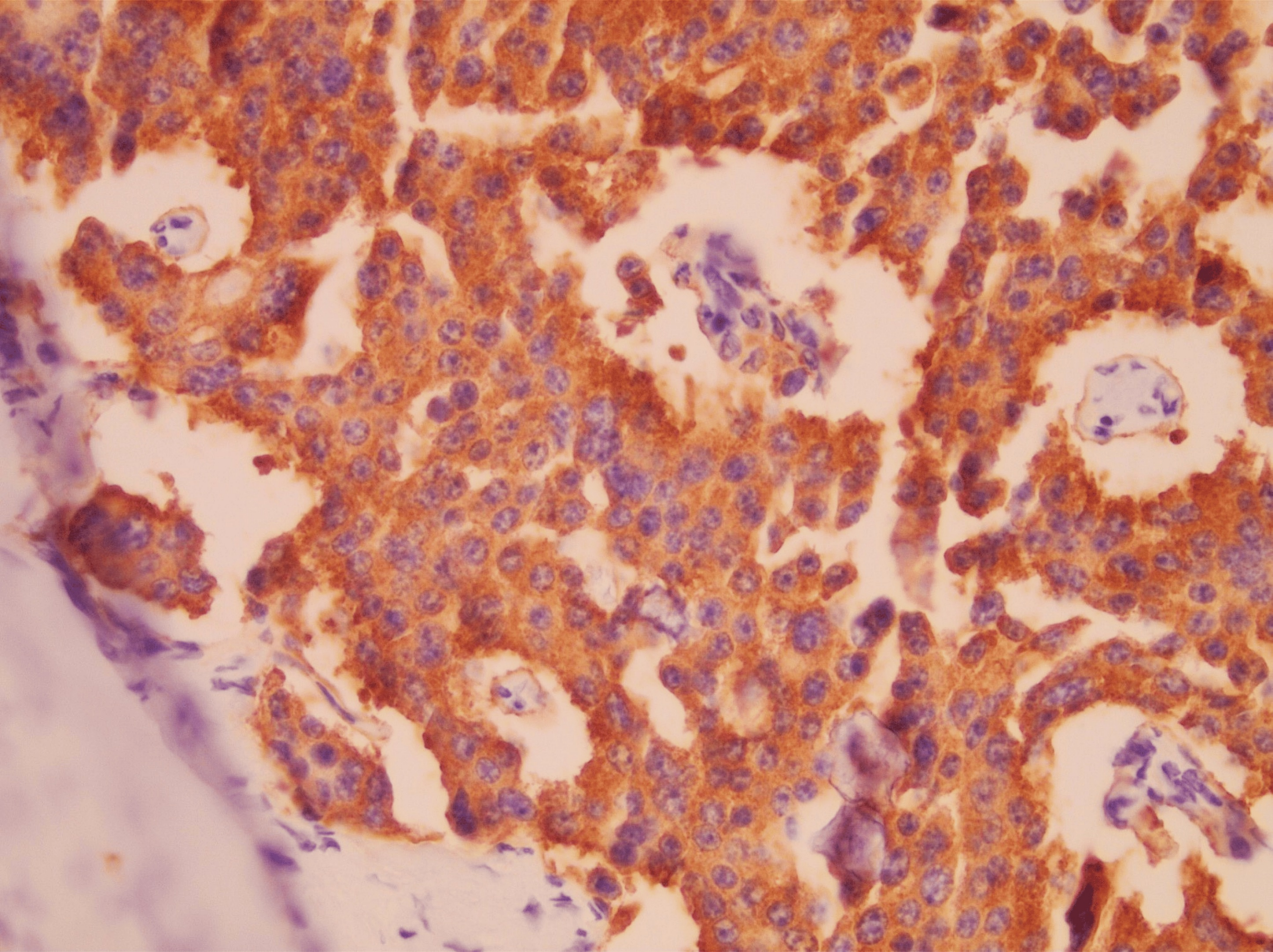
Synaptophysin immunohistochemical staining in well-differentiated neuroendocrine tumor Immunohistochemical staining for synaptophysin demonstrating diffuse cytoplasmic positivity in tumor cells, supporting neuroendocrine differentiation. The tumor cells show uniform morphology with round nuclei and finely stippled chromatin. Original magnification ×400.

Pathologic examination of the appendix showed partial fibrous obliteration, with one lymph node negative for malignancy and no dysplasia or malignancy identified. Additional proximal and distal small bowel margins showed focal ischemic changes without dysplasia or malignancy. Examination of the resected small bowel with mesenteric mass demonstrated mesenteric lymph nodes positive for metastatic well-differentiated neuroendocrine tumor, grade 1, with extranodal extension and lymphatic space invasion in several foci, along with associated focal ischemic changes in the small bowel (Figure 2). Immunohistochemical staining showed tumor cells positive for synaptophysin, chromogranin, CD56, and CK AE1/AE3. The Ki-67 proliferation index was less than 1% (Figure 3), supporting the diagnosis of a grade 1 well-differentiated neuroendocrine tumor (Figure 4 and Figure 5).

Postoperatively, the patient remained hemodynamically stable and improved clinically with bowel rest, intravenous fluids, analgesia, ambulation, and gradual diet advancement. He recovered without major postoperative complications and did not exhibit clinical features suggestive of carcinoid syndrome during hospitalization.

## Discussion

This case is notable because a biologically low-grade NET presented not as a functional endocrine syndrome but as a closed-loop small bowel obstruction with strangulation and ischemic bowel requiring emergency laparotomy. This presentation is clinically important and reflects the unique locoregional behavior of small bowel NETs. Even low-grade tumors may produce mesenteric nodal metastases and fibrosis severe enough to generate substantial mechanical and vascular consequences out of proportion to their proliferative index [4-10]. The case is particularly instructive because the patient had several months of intermittent abdominal symptoms before abrupt progression to a closed-loop obstruction, illustrating how the diagnosis may be missed until an emergency presentation occurs.

Carcinoid syndrome is absent in many patients with localized or regionally metastatic small bowel NETs and is more commonly associated with hormonally active tumors whose vasoactive products escape hepatic metabolism, particularly in the setting of liver metastases [5,13,14]. In contrast, many patients develop prolonged nonspecific symptoms such as vague abdominal pain, cramping, intermittent obstructive symptoms, altered bowel habits, or weight loss before eventually presenting with obstruction or ischemia [4-10]. In this patient, the three-month history of intermittent abdominal pain likely reflected evolving intermittent tethering or partial obstruction before abrupt progression to a closed-loop event.

The radiologic and operative findings in this case strongly support mesenteric fibrotic disease as the driver of obstruction. On CT, the mesenteric mass was located at the point of transition, with converging mesenteric vessels and associated bowel wall thickening concerning for ischemia. Intraoperatively, the bowel was tightly incarcerated and tethered by a stellate mesenteric mass, which is a classic gross correlate of the desmoplastic reaction described in small bowel NETs [7-10]. Histopathology then confirmed a metastatic well-differentiated NET in mesenteric lymph nodes with extranodal extension and lymphatic invasion. The presence of focal ischemic changes in the small bowel provides a coherent clinicopathologic explanation for the patient’s surgical emergency.

An important nuance is that the pathology did not explicitly identify a primary NET within the resected bowel itself. Instead, the malignancy was documented in mesenteric lymph nodes. Nevertheless, the overall radiologic, operative, and pathologic picture strongly suggests an occult small bowel primary, given the known biology of these tumors and their tendency to be small, multifocal, and sometimes difficult to identify grossly or on limited pathologic sampling [6,15].

## Conclusions

Small bowel NETs may remain clinically silent for months, yet first present as a surgical emergency. This case demonstrates that even a grade 1 metastatic well-differentiated NET can cause closed-loop small bowel obstruction, strangulation, and ischemic bowel through mesenteric nodal metastatic disease and desmoplastic tethering, even in the absence of carcinoid syndrome. In patients with a virgin abdomen, chronic intermittent abdominal pain, weight loss, and a mesenteric mass on CT imaging, NETs should remain in the differential diagnosis.
